# PUMA mediates the combinational therapy of 5-FU and NVP-BEZ235 in colon cancer

**DOI:** 10.18632/oncotarget.3775

**Published:** 2015-05-02

**Authors:** Huanan Wang, Lingling Zhang, Xu Yang, Yipeng Jin, Shimin Pei, Di Zhang, Hong Zhang, Bin Zhou, Yingjie Zhang, Degui Lin

**Affiliations:** ^1^ The Clinical Department, College of Veterinary Medicine, China Agricultural University, Beijing, China; ^2^ Department of Molecular Medicine, State Key Laboratory of Chemo/Biosensing and Chemometrics, College of Biology, Hunan University, Changsha, China; ^3^ Department of Biology, The Chinese University of Hong Kong, Shatin, New Territories, Hong Kong, China; ^4^ Department of Veterinary Medicine, College of Animal Sciences, Zhejiang University, Hangzhou, China

**Keywords:** colon cancer, NVP-BEZ235, PUMA, Akt, p53

## Abstract

Colon cancer is the third most common cancer in humans which has a high mortality rate, and 5-Fluorouracil (5-FU) is one of the most widely used drugs in colon cancer therapy. However, acquired chemoresistance is becoming the major challenges for patients, and the molecular mechanism underlying the development of 5-FU resistance is still poorly understood. In this study, a newly designed therapy in combination with 5-FU and NVP-BEZ235 in colon cancer cells (HCT-116 and RKO) was established, to investigate the mechanism of 5-FU resistance and optimize drug therapy to improve outcome for patients. Our results show 5-FU induced cell apoptosis through p53/PUMA pathway, with aberrant Akt activation, which may well explain the mechanism of 5-FU resistance. NVP-BEZ235 effectively up-regulated PUMA expression, mainly through inactivation of PI3K/Akt and activation of FOXO3a, leading to cell apoptosis even in the p53^−/−^ HCT-116 cells. Combination treatment of 5-FU and NVP-BEZ235 further increased cell apoptosis in a PUMA/Bax dependent manner. Moreover, significantly enhanced anti-tumor effects were observed in combination treatment *in vivo*. Together, these results demonstrated that the combination treatment of 5-FU and NVP-BEZ235 caused PUMA-dependent tumor suppression both *in vitro* and *in vivo*, which may promise a more effective strategy for colon cancer therapy.

## INTRODUCTION

Colorectal cancer (CRC) is the third most common cancer in humans and has a high mortality rate. In the year 2012, 103 170 new cases of colon cancer were diagnosed and 51 690 deaths occurred from colorectal cancer in the United States [1]. The five-year survival rate for metastatic colon cancer is less than 10%. Many therapeutic approaches have been used for the treatment of colon cancer, including chemotherapy, radiotherapy, targeted therapy, and immune therapy. 5-Fluorouracil (5-FU) is the most commonly used drug in colon cancer therapy with the capability to induce apoptosis in normal and tumor intestinal cells [2]. However, its response rate is less than 60%, even in combination with other chemotherapeutic agents [3]. Therefore, a big issue in the clinical application of 5-FU is drug resistance [4, 5]. Some researchers believe that 5-FU resistance has relationship with the mutation of drug target genes and the change of the expression levels of p53, bax, Bcl-2, and Bcl-xL [6]. But unfortunately, the precise molecular mechanism for 5-FU resistance in colon cancer remains elusive.

PUMA (p53 up-regulated modulator of apoptosis) is a BH3-only Bcl-2 family proteins, also known as bbc3 (Bcl-2 binding component 3), was first identified as a p53 downstream target [7–9]. It is localized in the mitochondria and induces apoptosis by activating Bax directly or indirectly which leads to mitochondrial outer membrane permeabilization (MOMP) [10, 11]. MOMP can trigger a cascade of downstream events to initiate apoptosis, including the release of proapoptotic factors such as SMAC, AIF, cytochrome c and the activation of caspase cascade [12]. The activation of PUMA by DNA damage is dependent on p53 and is mediated by the direct binding of p53 to the PUMA promoter region [8, 9, 13]. PUMA can also be induced via p53-independent manner in response to serum withdrawal as well as treatments with dexamethasone, tunicamycin and thapsigargin (TG) [14]. FOXO3a and p73 have been established as direct transcriptional regulators of PUMA [15, 16]. Therefore, PUMA plays an essential role in p53-dependent and -independent apoptotic pathways [17] and cells lacking PUMA are resistant to several death stimuli [18, 19]. Many studies have shown that PUMA is critical in colon cancer. Activation of PUMA by drug treatment killed the colon cancer cells which will live longer when PUMA was deficient [20–23]. Recently, people reported that PUMA plays an important role in promoting the apoptosis of prostate cancer and lymphoid malignancies [24, 25]. Meantime, PUMA was also involved in the regulation of autophagy, neuro disease and iPSC (induced pluripotent stem cells) generation [26–28].

AKT, also referred as protein kinase B (PKB), is a serine/threonine kinase which has three isoforms to share a high degree of sequence homology. All the three members (AKT1, AKT2, and AKT3) belong to the AGC family of protein kinases [29]. Akt resides in the cytosol and is activated through recruitment to cell membranes by phosphoinsitide 3-kinase (PI3K) lipid products and phosphorylation of its Thr308 and Ser473 residues [30]. Once activated, Akt is able to promote cell survival and proliferation through phosphorylation and inactivation of key components in the apoptotic cascade [31, 32]. Mammalian target of rapamycin (mTOR) is also a serine/threonine protein kinase belonging to the PI3K-related kinase family, which regulates cell growth, proliferation, motility, protein synthesis, and transcription [33]. It is established that the PI3K/Akt/mTOR pathway makes a big contribution in many cancers, including colon cancer [34, 35]. Furthermore, activation of this pathway has been shown to decrease sensitivity to chemotherapeutics as well as to irradiation (IR) [36, 37], which leads to diminished treatment success. Therefore, disrupting the PI3K/Akt/mTOR pathway may be a potential way for cancer therapy, or at least helpful for increasing sensitivity to chemo and radio therapy. In fact, there is a novel orally available dual PI3K/mTOR inhibitor NVP-BEZ235, which is currently in phase1/2 clinical trials for advanced solid tumors as a chemotherapeutic drug [38].

Our previous study shows that PUMA promotes Bax translocation both by directly interacting with Bax and by competitive binding to Bcl-X_L_ during cancer cell apoptosis [11, 39]. While PUMA has also been shown to mediate 5-FU induced colon cancer cell apoptosis [13, 19] and function as a critical regulator of apoptosis in colorectal cancer cells [20–23]. However, little is known about the molecular mechanism for 5-FU resistance in colon cancer, and new methods should be identified for the therapy. A recent study showed that NVP-BEZ235 is effective on both PIK3CA-mutated and unmutated colon cancer cell lines [40]. Therefore, NVP-BEZ235 may soon be incorporated into the present treatment regime for colon cancer. In the present study, we investigated the effect and related mechanism of 5-FU, NVP-BEZ235 and their combination treatment in colon cancer cells (HCT-116 and RKO). We found that the combination treatment significantly increased cancer cell apoptosis via a PUMA-dependent manner both *in vitro* and *in vivo*.

## RESULTS

### Combination treatment of 5-FU and NVP-BEZ235 dramatically suppressed cell proliferation and induced apoptosis

**Figure 1:**
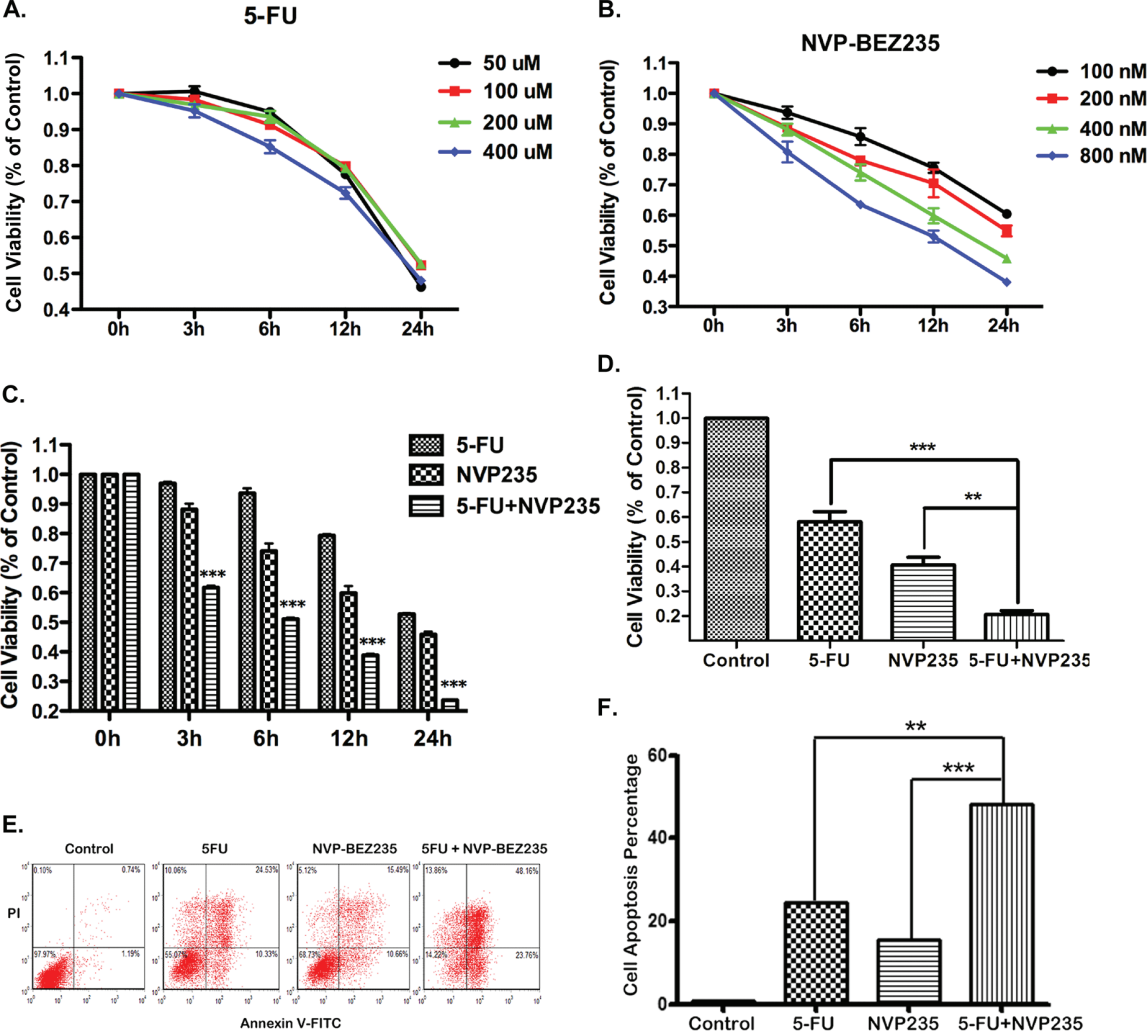
Efffects of 5-FU, NVP-BEZ235 or their combination on cell viability and apoptosis **A–D.** Cells viability was analyzed using Cell Counting Kit-8 at 0, 3, 6, 12 and 24 hours after (A) 50, 100, 200, or 400 uM 5-FU treatment, (B) 100, 200, 400, or 800 nM NVP-BEZ235 treatment, (C) the combination treatment of 200 uM 5-FU and 400 nM NVP-BEZ235 in HCT-116 cells, (D) Cell viability was detected in RKO cells after 24 hours combination treatment. **E.** HCT-116 cells apoptosis was analyzed using FACS technique at 12 hours after the treatment of 200 uM 5-FU, or 400 nM NVP-BEZ235, or their combination. **F.** The percentage of apoptotic cells were used to calculate. Data represent the mean ± S.D. of four independent experiments.

To establish a proper dose of 5-FU and NVP-BEZ25 in our system, HCT-116 cells were treated with various doses of 5-FU or NVP-BEZ235 respectively. Cell viability was analyzed using Cell Counting Kit-8 after 0, 3, 6, 12 and 24 hours treatment. Cells show a decrease in viability with increasing dose, and a correlation was observed with posttreatment period (Figure 1A and 1B), which indicates that the decrease of cell viability treated with 5-FU or NVP-BEZ235 is dose and time dependent. However, some different effects were seen between these two drugs. NVP-BEZ235 shows clear dose and time dependency, but for 5-FU, cell viability just has small fluctuation among different doses stimulation. To evaluate the efficacy of combination treatment, HCT-116 or RKO cells were exposed to either 200 uM 5-FU or 400 nM NVP-BEZ235 or in combination for 0, 3, 6, 12 and 24 hours. As shown in Figure 1C and 1D, cell viability has significant decrease after the combination treatment compared to single drug treatment, at all the time points.

Next, the status of cell apoptosis was evaluated by using FACS analysis. HCT-116 cells were treated with 200 uM 5-FU, 400 nM NVP-BEZ235 or their combination respectively. As shown in Figure 1E and 1F, the percentage of cell apoptosis increased evidently after 5-FU or NVP-BEZ235 stimulation, and this pro-apoptotic effect was potentiated when both drugs were used in combination compared to single therapy.

### 5-FU induced p53/PUMA dependent cell apoptosis, during which Akt was activated

**Figure 2:**
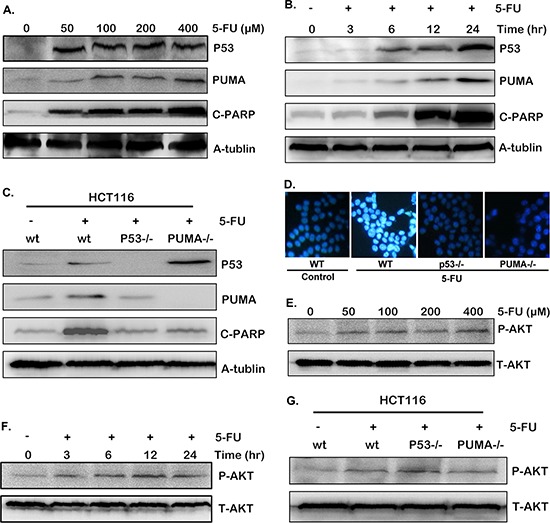
5-FU induces cell apoptosis through p53/PUMA pathway **A–C** and **E–G.** The expression of p53, PUMA, cleaved PARP and P-Akt(S473) were detected by western blotting in different conditions. (A) and (E) HCT-116 cells were treated with various doses of 5-FU for 24 hours. (B) and (F) HCT-116 cells were treated with 200 uM 5-FU, then harvested at different time points after stimulation. (C) and (G) Wild-type, p53^−/−^ or PUMA^−/−^ HCT-116 cells were treated with 200 uM 5-FU for 12 hours. (D) Hoechst 33342 morphological examination of apoptosis in wild-type, p53^−/−^ or PUMA^−/−^ HCT-116 cells. Cells were treated with 200 uM 5-FU and incubated for 12 hours, then stained with Hochest 333342, visualized under a Nikon fluorescent microscope (60 ×). Similar results were obtained from three independent experiments.

It is believed that p53/PUMA signaling pathway is very important in cell apoptosis and colon cancer therapy [13, 19–23]. To confirm this in our experiment model, the expression of p53 and PUMA were detected in cells exposed to 0, 50, 100, 200 and 400 uM 5-FU. After 24 hours treatment, p53 and PUMA expression was markedly up-regulated (Figure 2A). As a critical apoptotic marker, cleaved PARP also increased markedly (Figure 2A). Next, HCT-116 cells treated with 200 uM 5-FU was incubated for 0, 3, 6, 12 and 24 hours respectively. As shown in Figure 2B, the expression of both p53, PUMA and cleaved PRAP increased gradually over time.

To further confirm our results, parallel experiments have been carried out in the p53^−/−^ or PUMA^−/−^ HCT-116 cells. The results show increased PUMA expression as well as cleaved PARP after 5-FU stimulation in wild-type HCT-116 cells (Figure 2C), which were completely inhibited in the p53^−/−^ HCT-116 cells, (Figure 2C and S1A), implying p53 is required for 5-FU induced PUMA up-regulation and apoptosis. While in the PUMA^−/−^ HCT-116 cells, p53 expression was still up-regulated by 5-FU, but the cleaved PARP did not (Figure 2C and S1B), suggesting PUMA also contributed to 5-FU-induced apoptosis.

To observe cell apoptosis directly, morphological examination was performed with Hoechst 33342 staining. Chromatin condensation was observed in wild-type HCT-116 cells 12 hours after 5-FU treatment, which was almost completely restored in p53^−/−^ and PUMA^−/−^ HCT-116 cells (Figure 2D). Taken together, these results revealed that both p53 and PUMA are indispensable in 5-FU induced colon cancer cell apoptosis.

Akt activation is a sensitive marker for cancer cell growth, including colon cancer [34, 35]. So the phosphorylation of Akt was investigated in our experiment model. In theory, Akt is supposed to be inactivated after 5-FU treatment because of cell apoptosis. However, an opposite result was observed. Akt phosphorylation increased obviously after various doses of 5-FU treatment (Figure 2E), implying the existence of Akt survival pathway. Similar result was obtained after different time points of treatment (Figure 2F), even in the p53^−/−^ and PUMA^−/−^ HCT-116 cells (Figure 2G). The activation of Akt/mTOR pathway decreased sensitivity to chemotherapeutics as well as to irradiation (IR) [36, 37]. Therefore, this aberrant Akt activation indicates potential mechanism of 5-FU resistance in our system.

### NVP-BEZ235 up-regulated p53-independent PUMA expression

**Figure 3:**
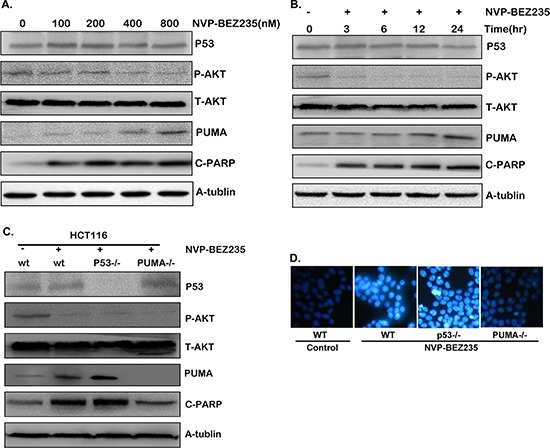
Cell apoptosis induced by NVP-BEZ235 is dependent on Akt/mTOR/PUMA pathway, not on p53 **A–C.** Western blotting showing the expression of P-Akt, p53, PUMA and cleaved PARP after (A) various doses of NVP-BEZ235 for 24 hours (B) 400 nM NVP-BEZ235 for different time stimulation in HCT-116 cells (C) 400 nM NVP-BEZ235 for 12 hours in wild-type, p53^−/−^ or PUMA^−/−^ HCT-116 cells. (D) Hoechst 33342 morphological examination of apoptosis in wild-type, p53^−/−^ or PUMA^−/−^ HCT-116 cells after the treatment of 400 nM NVP-BEZ235 for 12 hours. Similar results were obtained from three independent experiments.

Since 5-FU induced Akt activation may be the potential mechanism for 5-FU resistance, inhibiting Akt and its downstream pathways might increase the sensitivity of 5-FU treatment and further induce cancer cell apoptosis. To verify our hypothesis, HCT-116 cells were treated with various doses of NVP-BEZ235, or different time. As a result, Akt phosphorylation level declined distinctly (Figure 3A and 3B), which indicates the inhibition of Akt activation. Meantime, p53 expression had no change compared with that of the control group (Figure 3A and 3B). An interesting thing is PUMA expression was up-regulated by the NVP-BEZ235, company with increased cleaved PARP (Figure 3A and 3B). These results suggested that NVP-BEZ235 up-regulated PUMA expression and induced apoptosis by inhibiting Akt/mTOR pathway, which is in a p53-independent manner.

To further confirm our result, parallel experiments has been performed in the p53^−/−^ or PUMA^−/−^ HCT-116 cells. As a result, PUMA expression and cleaved PARP increased evidently along with Akt phosphorylation dropping in the p53^−/−^ HCT-116 cells (Figure 3C and S2A). However, in PUMA^−/−^ HCT-116 cells, cleaved PARP did not increase in spite of Akt was inactivated (Figure 3C and S2B). Furthermore, chromatin condensation was observed in both wild-type and p53^−/−^ HCT-116 cells after NVP-BEZ235 treatment, which did not occur in PUMA^−/−^ HCT-116 cells (Figure 3D). Taken together, these results indicated that PUMA is indispensable during NVP-BEZ235 induced colon cancer cell apoptosis, while p53 is not essential in this process.

### PI3K/Akt/FOXO3a pathway plays a major role in NVP-BEZ235-induced PUMA up-regulation

**Figure 4:**
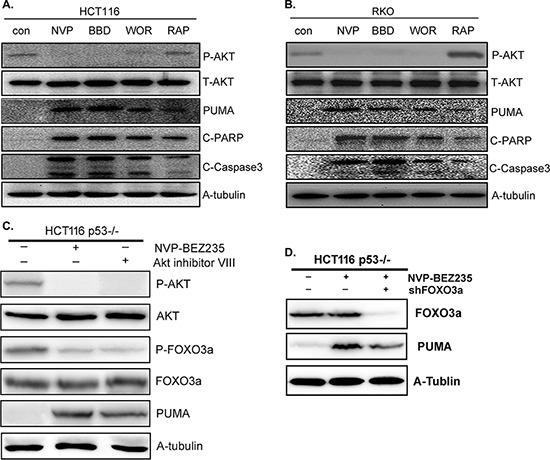
The effect of NVP-BEZ235, NVP-BBD130, Wortmannin, Rapamycin or Akt inhibitor VIII on PUMA expression and apoptosis **A–C.** The expression of P-Akt(S473), PUMA, P-FOXO3a, cleaved PARP and Caspase3 were detected after the treatment of 400 nM NVP-BEZ235, 400 nM NVP-BBD130, 250 nM Wortmannin, 50 nM Rapamycin or 5 uM Akt inhibitor VIII in (A) HCT-116 cells, (B) RKO cells. (C) p53^−/−^ HCT-116 cells. (D) FOXO3a and PUMA were detected in p53^−/−^ HCT-116 cells after the treatment of NVP-BEZ235, with FOXO3a deletion or not. Similar results were obtained from three independent experiments.

As p53 is not essential in NVP-BEZ235 induced apoptosis, and NVP-BEZ235 is a dual PI3K/mTOR inhibitor, PI3K, Akt, or mTOR may play a critical role in this process. To further study the molecular mechanism and clarify which one is dominant, another PI3K/mTOR dual inhibitor NVP-BBD130, PI3K inhibitor Wortmannin, and mTOR inhibitor Rapamycin (Sirolimus) were used to treat both HCT-116 and RKO cells. As shown in Figure 4A and 4B, NVP-BBD130 had an identical effect on decreasing Akt phosphorylation, up-regulating the expression of PUMA, cleaved PARP and Caspase3 as that of NVP-BEZ235. Wortmannin also effectively inhibited Akt phosphorylation, increased PUMA expression and apoptosis, although not so markedly as that of NVP-BEZ235. However, Rapamycin just had a minor effect on PUMA up-regulation and apoptosis (Figure 4A and 4B). These results suggested that PI3K/Akt plays a dominant role, whereas mTOR has a minor role in this process. Of course, the PI3K/mTOR dual inhibitior NVP-BEZ235 and NVP-BBD130 are superior to PI3K or mTOR single inhibitor to induce apoptosis.

As a direct transcriptional regulator of PUMA and downstream target of Akt [15], the status of FOXO3a was also detected in p53^−/−^ HCT-116 cells in the presence of Akt inhibitor VIII. The result shows that Akt blockade resulted in decreased phosphorylation of FOXO3a, indicating the activation of FOXO3a. In addition, and Akt inhibitor VIII had almost the same function as that of NVP-BEZ235 on PUMA up-regulation (Figure 4C). Next, FOXO3a was deleted by shFOXO3a in p53^−/−^ HCT-116 cells to evaluate its function. As shown in Figure 4D, FOXO3a deletion evidently suppressed PUMA up-regulation induced by NVP-BEZ235. Taken together, these results indicated that PI3K/Akt/FOXO3a plays a dominant role during NVP-BEZ235 induced PUMA up-regulation and apoptosis, while mTOR plays a minor role in this process.

### PUMA is indispensible in 5-FU and NVP-BEZ235 combination treatment induced colon cancer cell apoptosis

**Figure 5:**
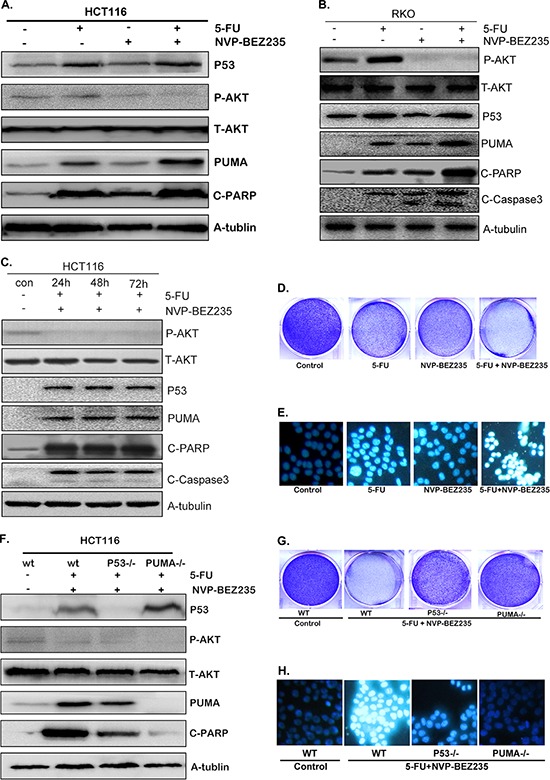
Combination treatment induced PUMA-dependent apoptosis **A–C.** Western blotting showing the expression of p53, P-Akt(S473), PUMA, cleaved PARP and Caspase3 after the treatment of 200 uM 5-FU, 400 nM NVP-BEZ235 or their combination for (A) 12 hours in HCT-116 cells, (B) 12 hours in RKO cells, (C) 24, 48 or 72 hours in HCT-116 cells. **(D** and **G).** Colony formation of HCT-116 cells. Cells were treated with different drugs for 24 hours, followed with crystal violet staining of attached cells at 14 days. (D) 200 uM 5-FU, 400 nM NVP-BEZ235, or their combination in wild-type HCT-116 cells, (G) combination treatment in wild-type, p53^−/−^ or PUMA^−/−^ HCT-116 cells. **E.** and **H.** Hoechst 33342 morphological examination of apoptosis in HCT-116 cells, (E) treated with 200 uM 5-FU, 400 nM NVP-BEZ235, or their combination for 12 hours in wild-type HCT-116 cells, (H) combination treatment for 12 hours in wild-type, p53^−/−^ or PUMA^−/−^ HCT-116 cells. **F.** The expression of p53, P-Akt, PUMA and cleaved PARP were detected in wild-type, p53^−/−^ or PUMA^−/−^ HCT-116 cells. Similar results were obtained from three independent experiments.

To address the effects of combination treatment of 5-FU and NVP-BEZ235 on PUMA expression and cell apoptosis, HCT-116 and RKO cells were treated with 200 uM 5-FU, 400 nM NVP-BEZ235 or their combination for 12 hours. As shown in Figure 5A and 5B, the combination treatment enhanced p53 expression but decreased Akt phosphorylation significantly. The exciting finding is that combination treatment had the synergistic effect on PUMA expression, cleaved PARP and Caspase3 compared with that of single drug treatment (Figure 5A and 5B). Furthermore, the analogous results were obtained in HCT-116 cells after long time combination treatment (Figure 5C). Consistently, the results of crystal violet and Hoechst 33342 staining also show synergistic effects of combination treatment on cell apoptosis (Figure 5D and 5E).

Additional experiments were performed in the p53^−/−^ or PUMA^−/−^ HCT-116 cells. As shown in Figure 5F and S3, combination treatment decreased Akt phosphorylation markedly in both wild-type, p53^−/−^ and PUMA^−/−^ HCT-116 cells. PUMA expression and cleaved PARP still increased evidently in p53^−/−^ cells, although not so strongly as that in wild-type cells (Figure 5F and S3B). However, in PUMA^−/−^ cells, cleaved PARP did not increase at all (Figure 5F and S3C), which was further confirmed by the crystal violet and Hoechst 33342 staining results (Figure 5G and 5H), indicating that PUMA is indispensible in combined treatment induced cell apoptosis.

### The combination treatment induced colon cancer cell apoptosis through PUMA/Bax pathway

**Figure 6:**
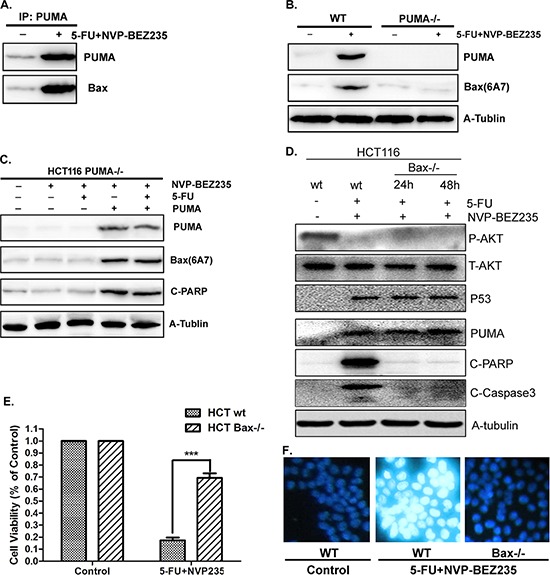
PUMA/Bax pathway is required for combination treatment induced apoptosis **A.** The interaction between PUMA and Bax was detected by Co-IP. Co-immunoprecipitation with an anti-PUMA antibody was used to pull down PUMA, western blotting for Bax shows the amount of Bax binding to PUMA. **B.** PUMA and activated Bax were detected in wide-type or PUMA^−/−^ HCT-116 cells after combination treatment. **C.** Transfecting PUMA into PUMA^−/−^ HCT-116 cells or not, western blot to check the expression of PUMA, activated Bax and cleaved PARP. **D.** P-Akt(S473), p53, PUMA, cleaved PARP and Caspase3 were detected in wild-type or Bax^−/−^ HCT-116 cells after combination treatment for 24 or 48 hours. **E.** Cell viability was analyzed by CCK-8 in wild-type or Bax^−/−^ HCT-116 cells after combination treatment for 48 hours. **F.** Hoechst 33342 morphological examination of apoptosis in in wild-type or Bax^−/−^ HCT-116 cells after combination treatment for 12 hours. Similar results were obtained from three independent experiments.

Our previous study demonstrated PUMA plays pro-apoptotic role by activating Bax directly and indirectly [11, 39]. Here, the relation between PUMA and Bax was investigated in HCT-116 cells with the combination treatment of 5-FU and NVP-BEZ235. As a result, there is a direct interaction between PUMA and Bax, which increased significantly after treatment (Figure 6A). Next, a conformation-specific anti-Bax monoclonal antibody (6A7) was used to detect the activated/pro-apoptotic form of Bax. The result shows that the combination treatment effectively activated Bax in wild-type HCT-116 cells, which did not occur in PUMA^−/−^ HCT-116 cells (Figure 6B). Furthermore, over-expressed PUMA in PUMA^−/−^ HCT-116 cells almost completely restored Bax activation and apoptosis induced by the combination treatment (Figure 6C). These results indicated that PUMA is an upstream regulator which is required for Bax activation.

To address the essential role of Bax activation in PUMA-mediated apoptosis, wild-type or Bax^−/−^ HCT-116 cells were treated with 5-FU and NVP-BEZ235 for 12, 24 or 48 hours. P53 and PUMA expression increased while phosphorylated Akt decreased in both wild-type and Bax^−/−^ cells after stimulation (Figure 6D and S4). Cleaved PARP and Caspase3 increased in wild-type treated cells, however, had no significant change in Bax^−/−^ cells (Figure 6D and S4). Consistent result was observed from cell viability analysis (Figure 6E). In addition, the result of Hoechst 33342 staining shows that chromatin condensation was blocked in Bax^−/−^ HCT-116 cells (Figure 6F). Therefore, PUMA mediated colon cancer cell apoptosis by activating Bax after combination treatment.

### The combination treatment caused much more tumor suppression *in vivo*

**Figure 7:**
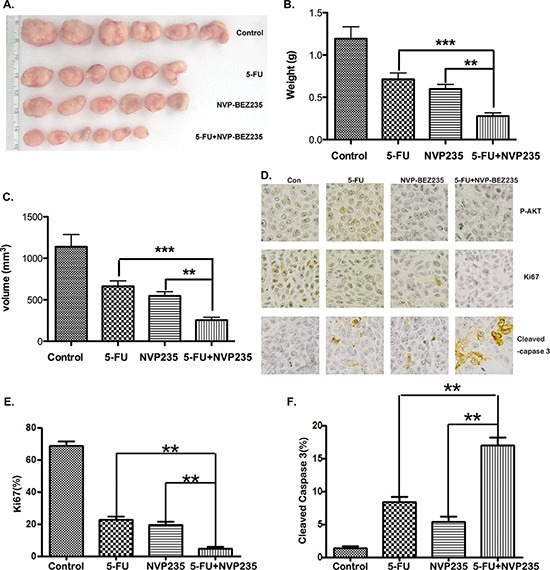
The antitumor effect of combination treatment *in vivo* **A–C.** Nude mice were injected s.c. with 1 × 10^6^ HCT-116 cells. Once the tumor was measurable, mice were treated daily with 5-FU at 25 mg/kg by i.p. injection, or 40 mg/kg NVP-BEZ235 by oral gavage, or their combination for 15 consecutive days. (A) Representative tumors at the end of the experiment, (B) tumors weight, (C) tumor volume at indicated time points after treatment was calculated. Data represent the mean ± S.D. of four independent experiments. **D.** Paraffin-embedded sections of control or treated tumor tissues from mice were analyzed by P-Akt(S473), ki67 and active Caspase-3 IHC staining. **E.** and **F.** Quantitative analysis of ki67 and active Caspase-3 staining corresponding to the images in D. Data represent the mean ± S.D. of four independent experiments.

As combination treatment is superior to either 5-FU or NVP-BEZ235 single treatment on PUMA up-regulation and cell apoptosis *in vitro*, we expect it will further suppress tumor growth *in vivo*. To confirm it, HCT-116 cells were injected subcutaneously into nude mice to establish xenograft tumors. Then the mice were treated with 25 mg/kg 5-FU by I.P. injection, or 40 mg/kg NVP-BEZ235 by oral gavage, or their combination for 15 consecutive days. As shown in Figure 7A, the combination treatment caused more significant tumor suppression compared to monotherapy. Quantitative analysis displays that the weight and volume of tumor in combination treatment group were less than 50% of that in 5-FU or NVP-BEZ235 single treatment group (Figure 7B and 7C). Immunohistochemistry staining of P-Akt, ki67 and cleaved Caspase-3 further confirmed that tumor growth was synergistically inhibited, with increasing apoptosis in combination treatment group (Figure 7D–7F).

## DISCUSSION

5-FU only had a single effective rate of 24%, and approximately 31% as the first-line drug of clinic chemotherapy on colon cancer, while the increase dosage could cause serious side-effects and drug resistance, which limited its dosage and therapeutic effect [41, 42]. Nevertheless, it is still the most common and widely used chemotherapeutic drug for the treatment of colon cancer. Therefore, a better understanding of the mechanism of the development of resistance to 5-Fu could definitely improve its therapeutic effect. A recent report shows that the mutation of drug target genes and the modification in the expression levels of p53, bax, Bcl-2, and Bcl-xL may be involved in the 5-FU resistance [6]. Here, our results suggest that aberrant Akt activation is a critical reason for 5-FU resistance in killing colon cancer cells. As shown in Figure 2E–2G, Akt activation enhanced evidently in wt, p53^−/−^ and PUMA^−/−^ HCT-116 cells after 5-FU stimulation, which well explained why there is almost no change on cell viability after increasing doses of 5-FU treatment (Figure 1A). Because as we all know, the PI3K/AKT/mTOR signaling pathways not only play an important role in normal cellular processes such as proliferation, survival, and differentiation [43], but also frequently deregulated in cancer cells and its constitutive activity strongly contributes to the oncogenic process [44, 45].

Since the activation of Akt/mTOR pathway decreased sensitivity to chemotherapeutics as well as to irradiation (IR) [36, 37], which was activated in our system, it may decrease the sensitivity of cells to 5-FU treatment. So we need to inactivate Akt and its downstream pathway, which can either increase the sensitivity of 5-FU therapy, or kill the cells directly, or both. Here, a novel orally available dual PI3K/mTOR inhibitor NVP-BEZ235, which is currently used in clinical trials with low side-effects, attracting our attention [38]. In the subsequent studies, NVP-BEZ235 or was proved to effectively inhibited Akt avtivation and induced colon cancer cell apoptosis (Figures 1, 3, and 4). NVP-BEZ235 induced cell apoptosis through different pathway from 5-FU, because it did not affect p53 expression in wt HCT-116 cells, and killed p53^−/−^ HCT-116 cells as wt cells (Figure 3), whereas p53 is required for 5-FU induced cell apoptosis (Figure 2). The most exciting finding is PUMA expression was up-regulated by NVP-BEZ235 treatment via a p53-independent way (Figure 3C), and it is required for both 5-FU and NVP-BEZ235 induced cell apoptosis (Figures 2C, 2D, 3C and 3D). These results indicated that PUMA built up a crosstalk between 5-FU-induced and NVP-BEZ235-induced apoptotic pathways.

As a PI3K/mTOR dual inhibitor, NVP-BEZ235 up-regulates PUMA expression and induces apoptosis through a complex pathway. To clarify it, different inhibitors including NVP-BBD130, Wortmannin, Rapamycin and Akt inhibitor VIII were used to compare with NVP-BEZ235 to illustrate the effect of PI3K, Akt or mTOR during NVP-BEZ235-induced apoptosis. We found that all the inhibitors, except Rapamycin, have identical effect on Akt inactivation, and increasing PUMA expression and apoptosis (Figure 4A–4C), whereas Rapamycin had no effect on Akt activity and only slightly increased PUMA expression and cell apoptosis, suggesting a minor role in this process (Figure 4A and 4B). FOXO3a was activated as a downstream target when Akt was inactivated (Figure 4C), which plays an essential role in PUMA regulation (Figure 4D). All these indicated PI3K/Akt/FOXO3a is the dominant pathway, and PI3K/Akt/mTOR is the subordinate pathway, to mediate NVP-BEZ235-induced PUMA up-regulation and apoptosis. Of course, we can not exclude other regulators, waiting there to be discovered in the process.

As we expected, combination treatment further increased PUMA expression and cell apoptosis (Figure 5A–5E), and PUMA is indispensible in this process (Figure 5F–5H). Akt inhibitor VIII was also used to treat the colon cancer cells in combination with 5-FU, and obviously increased the sensitivity of 5-FU therapy (Figure S3A), which further demonstrated the importance of Akt activation in resistance. In addition, PUMA is the direct upstream regulator for Bax activation (Figure 6A–6C), which plays an important role in cell apoptosis (Figure 6D–6F), indicating the combined treatment activated the mitochondrial apoptotic pathway. For the *in vivo* experiments, the combination treatment caused much more tumor suppression and apoptosis than that of single treatment of either 5-FU or NVP-BEZ235 (Figure 7), which further confirmed our conclusion.

**Figure 8:**
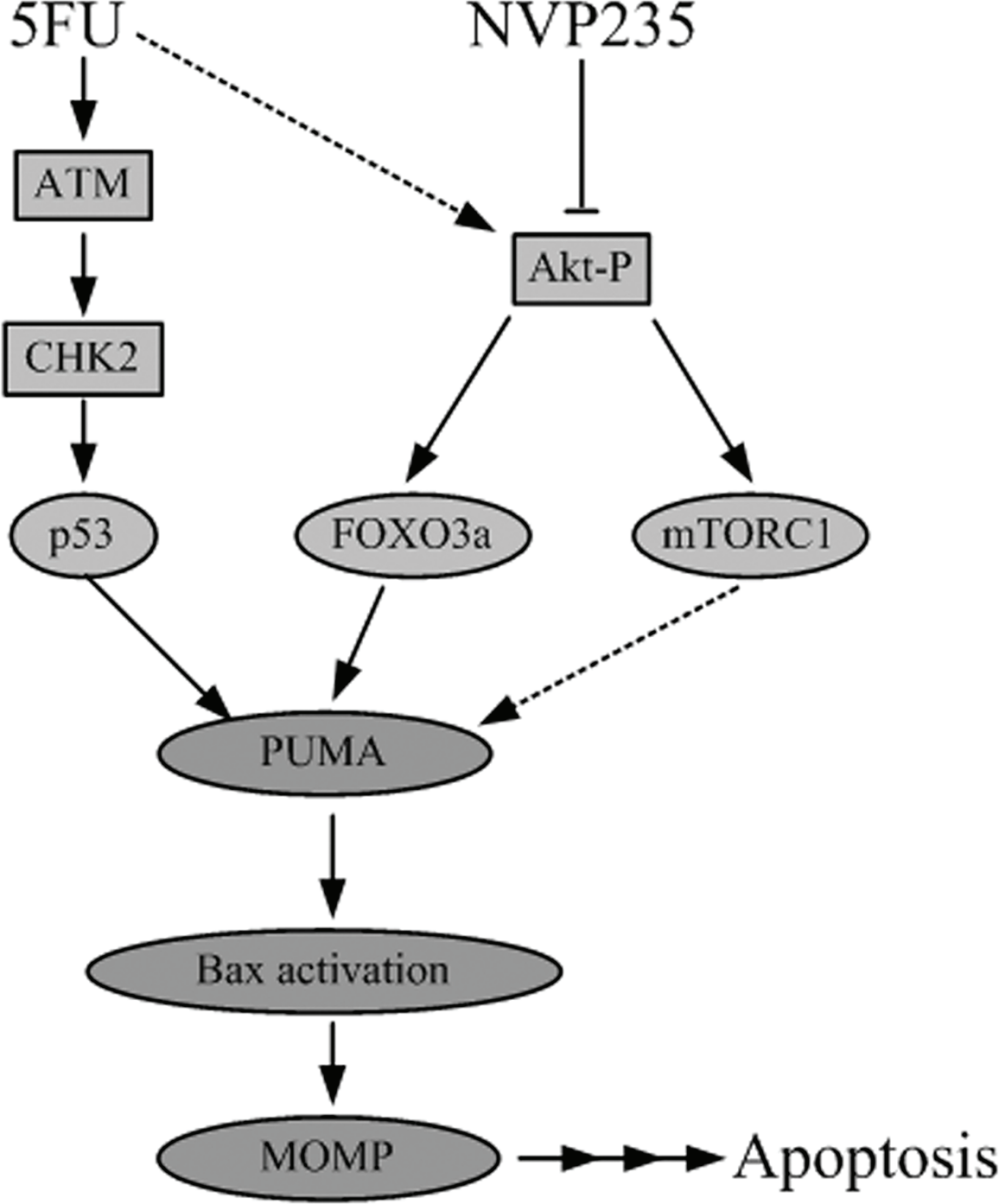
Schematic representation of 5-FU and NVP-BEZ235 induced apoptotic pathway

For the first time, our results demonstrated that 5-FU-induced Akt activation is the potential mechanism for its resistance. NVP-BEZ235 up-regulated PUMA expression and induced colon cancer cell apoptosis via a p53-independent, but an Akt/FOXO3a dependent way (Figure 8). Therefore, NVP-BEZ235 potentises the anti-tumor effect not only by inhibiting Akt survival pathway but also promoting cell apoptosis through PUMA/Bax pathway. These raise the possibility of combination treatment to develop a promising therapeutic strategy to enhance the effects of chemotherapy and improve clinical outcomes for colon cancer patients with a p53 mutation. Further studies are necessary to pinpoint whether Noxa or Bim, the classic downstreams of p53 and Akt, is required in this process. Meantime, more precise dose selection should be conducted in animal and clinical studies.

## MATERIALS AND METHODS

### Cell culture and treatments

The human colon cancer cell line (HCT-116), Human colon cancer cell line with p53, PUMA, or Bax null (HCT-116 p53^−/−^, HCT-116 PUMA^−/−^, or HCT-116 Bax^−/−^) were cultured in McCoy's 5A, the human colon cancer cell line (RKO) was cultured in Eagle's minimum essential medium (EMEM), supplemented with 10% fetal bovine serum (FBS), penicillin (100 units/ml), and streptomycin (100 mg/ml) in 5% CO2 at 37°C in humidified incubator. For treatment, various doses of 5-FU (50, 100, 200, or 400 uM), NVP-BEZ235 (100, 200, 400, or 800 nM), or their combination (200 uM 5-FU and 400 nM NVP-BEZ235) were added in the medium directly before detection. Transfections were performed with Lipofectamine™ 2000 reagent according to the manufacturer's protocol. The medium was replaced with fresh culture medium after 5 hours. Cells were examined at 24–48 hours after transfection.

### Antibodies and reagents

Primary antibodies against p53, Phospho-Akt(S473), total-AKT, PUMA, cleaved PARP, Ki67, and cleaved Caspase3 were purchased from cell signaling; alpha-tubulin antibody was from Santa Cruz Technologies. Lipofectamine™ Reagent was purchased from Invitrogen. HRP-conjugated anti-rabbit or anti-mouse secondary antibodies and an ECL-plus kit were from GE Healthcare. 5-FU was purchased from APP Pharmaceuticals and NVP-BEZ235 was purchased from LC Laboratories. The plasmid of expressing PUMA was kindly supplied by Jian Yu, Ph.D. [9]. The oligonucleotide for shFOXO3a was synthesized as 5′-CACCGACTCCGGGTCCAGCTCCACTTCAAGAGAGTGGAGCTGGACCCGGAGTTTT TTTG-3′. NVP-BBD130 was purchased from Axon Medchem. Wortmannin and Rapamycin (Sirolimus) were purchased from Selleck Chemicals. Other chemicals were mainly from Sigma.

### Cell viability and apoptosis assays

HCT-116 or RKO cells were cultured in 96-well microplate at a density of 5 × 10^3^ cells/well for 24 hours. The cells were then divided into several groups and treated with 5-FU, NVP-BEZ235, or their combination. Cell viability was assessed with CCK-8 (Dojindo Laboratories, Kumamoto, Japan) at 0, 3, 6, 12 or 24 hours post-treatment according to the manufacturer's instructions. OD_450_, the absorbance value at 450 nm, was read with a 96-well plate reader (DG5032, Hua dong, Nanjing, China), to determine the viability of the cells.

For analysis of apoptosis by nuclear staining, HCT-116 cells were cultured on the coverslip of a chamber, rinsed with phosphate-buffered saline (PBS) and then 500 ml DMEM containing 5 μg Hoechst 33342 was added in, incubated at 37°C with 5% CO2 for 15 minutes. Apoptosis was assessed through microscopic visualization of condensed chromatin and micronucleation.

### Flow cytometry

For Flow cytometric analysis (FACS analysis), Annexin-V-FITC conjugate and binding buffer were used as standard reagents. Flow cytometry was performed on a FACScanto flow cytometer (Becton Dickinson, Mountain View, CA, USA) with excitation at 488 nm. Fluorescent emission of FITC was measured at 515–545 nm and that of DNA-PI complexes at 564–606 nm. Cell debris was excluded from analysis by an appropriate forward light scatter threshold setting. Compensation was used wherever necessary.

### Western blotting

At the indicated time after drug treatment, cells were harvested and washed twice with ice-cold phosphate-buffered saline (PBS, PH 7.4), and lysed with ice-cold lysis buffer (50 mmol/L Tris HCl PH 8.0, 150 mmol/L NaCl, 1 × TritonX-100, 100 μg/ml PMSF) for 30 minutes on ice. The lysates were centrifuged at 12, 000 rpm for 5 minutes at 4°C, and the protein concentration was determined. Equivalent samples (30 μg protein extract was loaded on each lane) were subjected to SDS-PAGE on 10% gel. The proteins were then transferred onto PVDF membranes (GE Healthcare), and probed with indicated primary antibody. Primary antibody was detected by binding horseradish peroxidase (HRP)-conjugated anti-rabbit or anti-mouse secondary antibody with an ECL plus kit.

### Co-immunoprecipitation

After 5-FU and NVP-BEZ235 treatment, cells were harvested and washed twice with ice-cold phosphate-buffered saline (PBS, PH 7.4), and lysed with ice-cold lysis buffer (10 mmol HEPES PH 7.4, 150 mmol/L NaCl, 1% CHAPS, 1% protease inhibitors) for 30 minutes on ice. For immunoprecipitation (IP), about 4 μl of IP antibodies were added to 400 μl cell lysates. The mixtures were mixed on a rocker at ambient temperature for 2 hours. The immunocomplexes were captured by the addition of protein G/A-agarose (Roche Applied Sciences) mixed at 1:10 ratio, followed by incubation at ambient temperature for 1 hour. The beads were washed three times by PBS and then collected by centrifugation at 12000 rpm for 5 seconds. After the final wash, the beads were mixed with 60 μl of 2 × Laemmli sample buffer, heated at 100°C for 5 min, and analyzed by Western blotting.

### Xenograft mouse model and treatment

Female 5- to 6-week-old nude mice (Vital River, China) were housed in a sterile environment with micro isolator cages and allowed access to water and chow *ad libitum*. 1 × 10^6^ cells were resuspended in 100 ul of PBS (phosphate-buffered saline solution) and injected subcutaneously into the flanks of nude mice. Once the tumor was measurable, mice were treated daily with 5-FU at 25 mg/kg by i.p. injection, or 40 mg/kg NVP-BEZ235 by oral gavage, or their combination. NVP-BEZ235 was dissolved in NMP/PEG300 (1:9), solicated in NMP firstly, and then PEG300 was added till the final volume; 5-FU was supplied as a stock solution. Mice were treated for 7 days a week, and terminated after 15 days treatment. Tumor growth was monitored by calipers, and tumor volumes were calculated according to the formula 0.5 × length × width^2^. Mice were euthanized when tumors reached ~1.0 cm^3^ in size.

### Autopsy and histopathology

Animals were autopsied when the tumor reached to the maximal size and tissues were collected and examined. Experimental and control tissue samples were fixed in 10% neutral-buffered formalin 24 hours, and washed once with 1X PBS and then transferred into 70% ethanol and stored at 4°C. Tissues were processed by ethanol dehydration and embedded in paraffin by Leica according to standard protocols. Sections (5 um) were prepared for hematoxylin staining.

## SUPPLEMENTARY FIGURES



## Abbreviations

PUMA: p53 up-regulated modulator of apoptosis
Bax: Bcl-2-associated X protein
MOMP: mitochondrial outer membrane permeabilization
CCK-8: Cell Counting Kit-8
PARP: poly ADP-ribose polymerase
5-FU: 5-fluorouracil
mTOR: mechanistic target of rapamycin
