# Supplementary material for: PUMA mediates the combinational therapy of 5-FU and NVP-BEZ235 in colon cancer
Source: Oncotarget. 2015 May 2;6(16):14385–98. doi: 10.18632/oncotarget.3775 (PMC4546474; doi:10.18632/oncotarget.3775)
Supplement: Supplementary file 1 [file oncotarget-06-14385-s001.pdf]

## SUPPLEMENTARY FIGURES

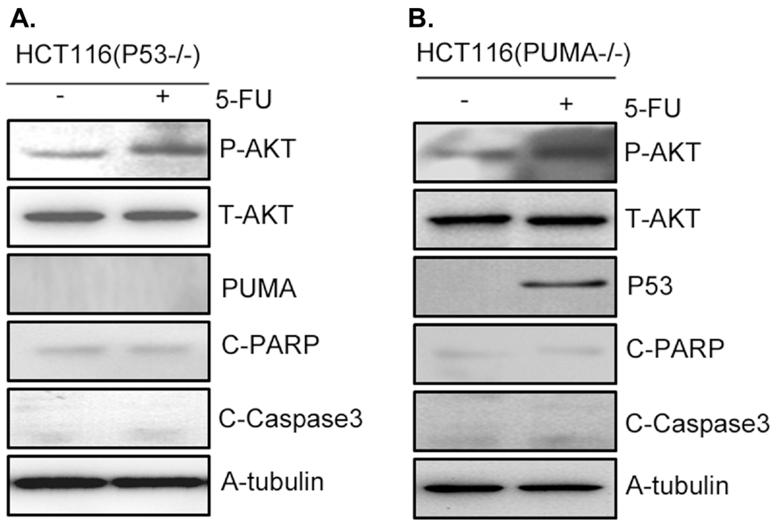


**Supplementary Figure S1: 5-FU induces cell apoptosis in p53$^{-/-}$ or PUMA$^{-/-}$ HCT-116 cells. A.** and **B.** Cells were treated with 200 uM 5-FU for 12 hours, then P-Akt(S473), p53, PUMA, cleaved PARP and Caspase3 were detected in (A) p53$^{-/-}$ HCT-116 cells and (B) PUMA$^{-/-}$ HCT-116 cells. Similar results were obtained from three independent experiments.

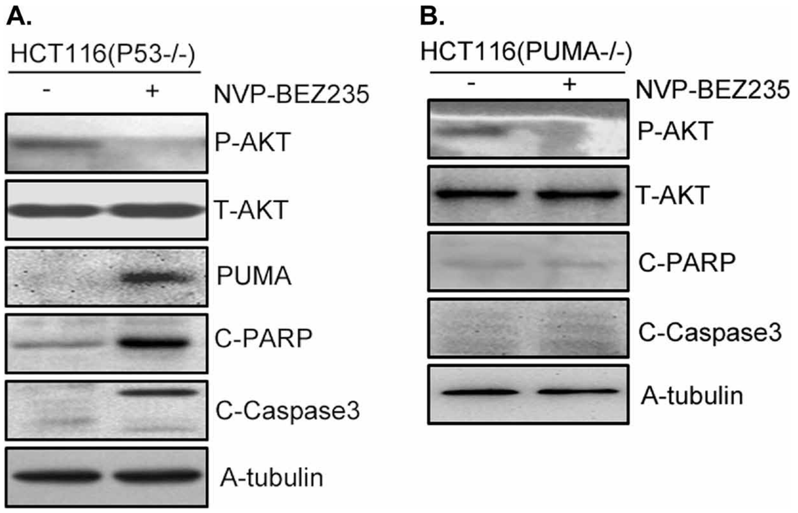


**Supplementary Figure S2: NVP-BEZ235 induces cell apoptosis in p53$^{-/-}$ or PUMA$^{-/-}$ HCT-116 cells. A.** and **B.** Cells were treated with 400 nM NVP-BEZ235 for 12 hours, then P-Akt(S473), PUMA, cleaved PARP and Caspase3 were detected in (A) p53$^{-/-}$ HCT-116 cells and (B) PUMA$^{-/-}$ HCT-116 cells. Similar results were obtained from three independent experiments.

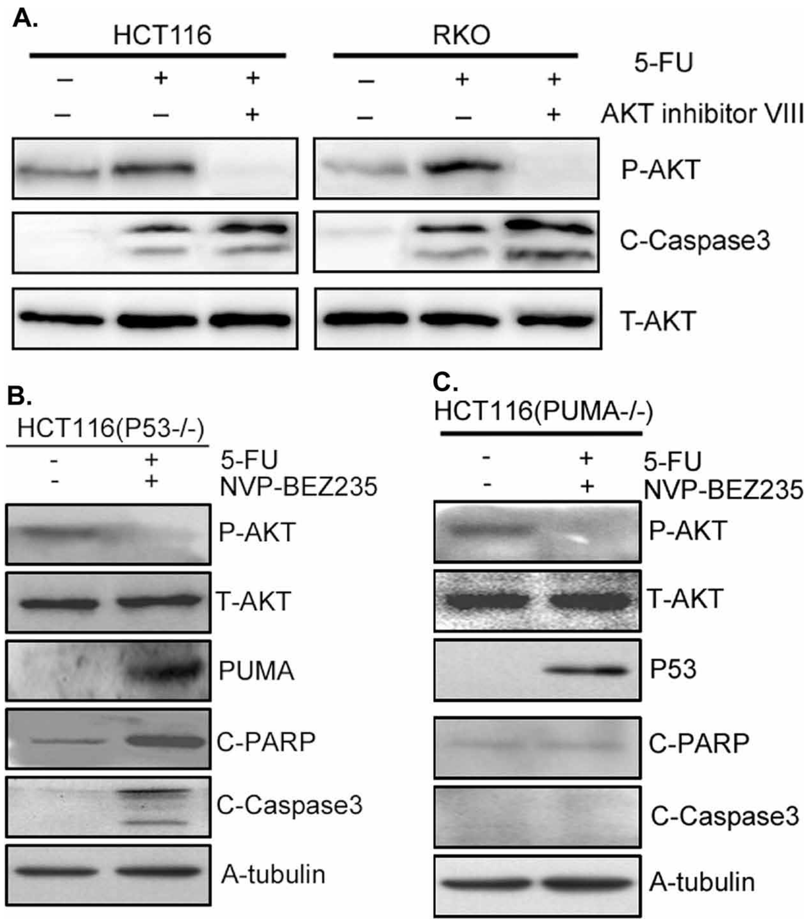


**Supplementary Figure S3: Combination treatment induces cell apoptosis in HCT-116 and RKO cells. A.** P-Akt(S473) and cleaved Caspase3 were detected in HCT-116 and RKO cells after the treatment of 200 uM 5-FU, or the combination of 200 uM 5-FU and 5 uM Akt inhibitor VIII. **B.** and **C.** Cells were treated with the combination of 200 uM 5-FU and 400 nM NVP-BEZ235 for 12 hours, then P-Akt(S473), p53, PUMA, cleaved PARP and Caspase3 were detected in (B) $p53^{-/-}$ HCT-116 cells and (C) $PUMA^{-/-}$ HCT-116 cells. Similar results were obtained from three independent experiments.

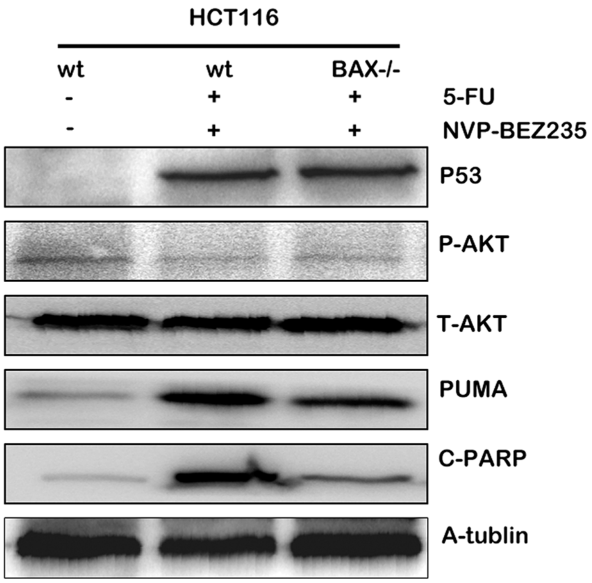


**Supplementary Figure S4: Combination treatment induces cell apoptosis in wild-type and Bax–/– HCT-116 cells.** P-Akt(S473), p53, PUMA and cleaved PARP were detected in wild-type and Bax$^{-/-}$ HCT-116 cells after combination treatment of 200 uM 5-FU and 400nM NVP-BEZ235 for 12 hours. Similar results were obtained from three independent experiments.
